# Comprehensive clinical evaluation of novel 4DCT‐based lung function imaging methods

**DOI:** 10.1002/acm2.70088

**Published:** 2025-04-08

**Authors:** Ehsan Golkar, Taindra Neupane, Lydia Wilson, Jennifer Kwak, Richard Castillo, Edward Castillo, Yevgeniy Vinogradskiy

**Affiliations:** ^1^ Department of Radiation Oncology Thomas Jefferson University Philadelphia USA; ^2^ Department of Radiology University of Colorado School of Medicine Aurora USA; ^3^ Department of Radiation Oncology Emory University Atlanta USA; ^4^ Department of Biomedical Engineering University of Texas at Austin Texas USA

**Keywords:** Lung cancer, lung functional avoidance, ventilation imaging, 4DCT‐ventilation

## Abstract

**Purpose:**

Methods have been developed that apply image processing to 4DCTs to generate 4DCT‐ventilation/perfusion lung imaging. Traditional methods for 4DCT‐ventilation rely on Hounsfield‐Unit (HU) density‐change methods and suffer from poor numerical robustness while not providing 4DCT‐perfusion data. The purpose of this work was to evaluate the clinical differences between classic HU‐based 4DCT‐ventilation approaches and novel 4DCT‐ventilation/perfusion approaches.

**Methods:**

Data from 63 lung cancer patients enrolled in a functional avoidance clinical trial were analyzed. 4DCT‐data were used to generate four lung‐function images: (1) classical HU‐based 4DCT‐ventilation (“4DCT‐vent‐HU”), and three novel, statistically robust methods: (2) 4DCT‐ventilation based on the Mass Conserving Volume Change (“4DCT‐vent‐MCVC”), (3) 4DCT‐ventilation using the Integrated Jacobian Formulation, and (4) 4DCT‐perfusion. A radiologist reviewed all images for ventilation/perfusion defects (scored as yes/no) and the scores for the novel approaches were compared to those of 4DCT‐vent‐HU using receiver operating characteristic (ROC) analysis. Functional contours were generated using thresholding methods, and the contours from the three novel 4DCT‐ventilation methods were compared against that from 4DCT‐vent‐HU (Dice similarity coefficients [DSC]). Functional mean lung dose (fMLD) and dose‐function metrics were compared against dose‐function metrics using 4DCT‐vent‐HU.

**Results:**

ROC analysis revealed accuracy in the range of 0.55 to 0.73 comparing radiologist interpretations of 4DCT‐vent‐HU against the three novel approaches. Average DSC values were 0.41 ± 0.19, 0.44 ± 0.16, and 0.42 ± 0.17 comparing 4DCT‐vent‐HU to 4DCT‐vent‐IJF, 4DCT‐vent‐MCVC, and 4DCT‐perf, respectively. All novel imaging methods showed significant differences (*p* < 0.01) in dose‐function metrics compared to those of 4DCT‐vent‐HU. 4DCT‐vent‐MCVC and 4DCT‐Perf depicted the smallest and largest differences from 4DCT‐vent‐HU in fMLD (3.51 ± 3.20 Gy and 5.90 ± 5.29 Gy, respectively).

**Conclusion:**

This is the first work to comprehensively compare novel 4DCT‐ventilation/perfusion methods against classical formulations. Our data show that significant differences between the 4DCT‐based functional imaging methods exist, suggesting that studies are needed to evaluate which methods provide the most robust clinical results.

## 1 INTRODUCTION

Patients with lung cancer often experience persistent respiratory difficulties, substantially impacting their quality of life.^1^ Traditional treatments for lung cancer include chemotherapy, radiation therapy, immunotherapy, and surgery. However, radiation‐induced pulmonary toxicity remains a concern for patients undergoing radiation therapy.^2^ Typical pulmonary toxicities include radiation pneumonitis, dyspnea (difficulty breathing), and decreased lung function.^3^ Pneumonitis, in particular, is potentially dangerous and can limit the treatment options for patients.^4^

Radiation treatment is designed to limit healthy lung doses (i.e., outside the tumor volume), such as the mean lung dose and the volume of the lung receiving ≥20 Gy (lung V20 Gy).^5^ Standard lung dose‐volume constraints assume homogeneous lung function,^6^ which may not reflect the true spatial heterogeneity in lung function observed in many patients.^7^ Studies show that most lung cancer patients have heterogeneous lung function, often due to the tumor and pre‐existing respiratory diseases.^8^

Functional avoidance thoracic radiotherapy aims to reduce pulmonary toxicity by designing radiation treatment plans that avoid functional portions of the lung, as defined by functional imaging modalities.^9^, ^10^ The functional avoidance approach hypothesizes that sparing functional lung regions from high radiation doses will reduce rates of pulmonary toxicity.^10^, ^11^ Among the various functional imaging modalities proposed for functional avoidance, 4‐dimensional computed tomography (4DCT) ventilation (4DCT‐ventilation) imaging is a promising technique to calculate 4DCT‐based lung ventilation maps.^7^, ^12^ 4DCT‐ventilation uses 4DCT data and advanced image processing techniques to generate lung ventilation maps.^13^ 4DCT‐ventilation is advantageous in radiation oncology as it uses routinely collected 4DCT imaging without requiring an additional imaging procedure.^7^, ^9^ The 4DCT‐ventilation technique has been validated against established modalities such as nuclear medicine single‐photon emission computed tomography (SPECT) ventilation,^14^ hyperpolarized helium or xenon MRI,^15^ and positron emission tomography (PET) perfusion imaging,^16^ showing promising correlation results.^17^

Studies have shown that dose‐function metrics (where the function is defined using 4DCT‐ventilation imaging) are more predictive of radiation toxicity than dose metrics alone.^9^, ^18^, ^19^ Clinical trials have investigated the use of 4DCT‐ventilation‐based functional avoidance in lung cancer patients.^10^, ^11^ By sparing functional lung regions, the rates of radiation pneumonitis were reduced compared to pneumonitis rates present with standard thoracic radiotherapy.^20^ Traditional functional avoidance studies (including clinical trials) have employed either the Hounsfield Unit (HU) or Jacobian‐based 4DCT‐ventilation image generation approaches. However, shortcomings of these methods have been noted including numerical instability, reduced imaging robustness, and an inability to generate 4DCT‐perfusion imaging information in addition to 4DCT‐ventilation.^17^, ^21^ To address these challenges, three novel 4DCT‐based lung function imaging methods were developed referred to as the Integrated Jacobian Formulation (4DCT‐vent‐IJF),^22^ Mass Conserving Volume Change (4DCT‐vent‐MCVC) formulation,^23^ and 4DCT‐based perfusion (4DCT‐perfusion).^24^ The 4DCT‐vent‐IJF, 4DCT‐vent‐MCVC, and 4DCT‐perfusion methods rely on generating lung function images that use deformable image registration (DIR) solutions that respect the spatial accuracy on the digital voxel grid and are based on DIR‐measured subregional volume change estimates acquired with quantifiable and controllable levels of uncertainty. The IJF, MCVC, and 4DCT‐perfusion methods have been shown to provide improved numerical stability, superior imaging robustness, perfusion information in addition to ventilation, and better agreement with other forms of lung‐function imaging compared to tradition IJF and HU‐based approaches.^22^, ^23^, ^25^ Although the improved image robustness and mathematical theory of the novel methods has been presented, there have been no studies that have evaluated the clinical image and functional avoidance differences between the novel methods and established forms of 4DCT‐ventilation imaging. The purpose of this study was therefore to quantitatively evaluate the differences between the novel, robust 4DCT‐based ventilation and perfusion methods against established 4DCT‐based imaging techniques.

## 2 METHODS

### 2.1 Patient cohort

A clinical trial was carried out at the University of Colorado, VA Eastern Colorado Health Care System in Aurora, CO (IRB #14‐1856), and Beaumont Health System in Royal Oak, MI (IRB #2016‐037) from April 2015 to December 2019. The trial was structured as a phase 2 trial, aimed at comparing the incidence of grade 2 or higher radiation pneumonitis against historical controls. The study enrolled 67 patients diagnosed with either small cell lung cancer (SCLC) or non‐small cell lung cancer (NSCLC). All participants were at least 18 years old and were scheduled to undergo curative‐intent chemotherapy and radiation therapy, with no restrictions on baseline patient performance status or pulmonary function tests (PFTs). Curative‐intent radiation therapy was defined as prescription doses ranging from 45 to 75 Gy. All selected patients were treated using standard fractionation schemes of 1.8 to 2.0 Gy per fraction. The trial incorporated a 4DCT‐ventilation imaging heterogeneity criterion, requiring both a quantitative 15% decrease (derived from prior studies^10^, ^26^, ^27^) in lung function near the tumor and a qualitative ventilation defect assessment by a radiation oncologist. The 4DCT‐ventilation imaging heterogeneity criterion was grounded in nuclear medicine concepts and aimed to identify lung regions in patients with heterogeneous function for targeted functional avoidance.^26^, ^28^ The quantitative 15% decrease in lung function was based on prior studies showing that a 15% decrease in lung function was most predictive of radiologist interpretation of lung function defects.^10^, ^25^, ^26^ The clinical parameters of the trial patient cohort have been previously described in detail.^10^ Patients enrolled on the trial had a median age of 65 years (range: 45–86 years), with 64.3% female and 35.7% male. The majority (82.1%) had NSCLC, while 17.9% had SCLC. Disease stages were distributed as follows: 3.6% stage I, 8.9% stage II, 76% stage III, and 10.7% stage IV. Radiation therapy was prescribed at a median dose of 60 Gy (range: 45–66 Gy) over a median of 30 fractions (range: 23–33).^29^ A total of 63 patient tumors were identified, with 43% located in the right upper lobe, 10% in the right middle lobe, 17% in the right lower lobe, 17% in the left upper lobe, and 13% in the left lower lobe.

As part of standard treatment planning each patient underwent a 4DCT scan. Additionally, as part of the trial, 25 patients underwent ventilation perfusion planar scan, and 30 patients underwent Single Photon Emission Computed Tomography (SPECT) ventilation/perfusion scans. Of the 67 patients enrolled on the clinical trial, 63 patients were selected for the current analysis, excluding those with a single lung or significant 4DCT artifacts.

### 2.2 4DCT‐ventilation‐perfusion image generation

**FIGURE 1:**
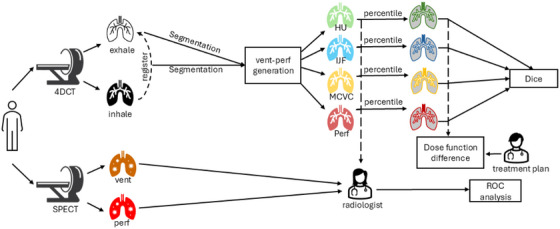
Schema of functional image generation and analysis. HU, Hounsfield‐Unit; IJF, Integrated Jacobian Formulation; MCVC, Mass Conserving Volume; Perf, perfusion; ROC, receiver operating characteristic; Vent, ventilation.

The overall study design is presented in Figure 1. The goal of the work was to evaluate the differences between established methods of generating 4DCT‐ventilation images^30^, ^31^ and the novel robust 4DCT‐ventilation and 4DCT‐perfusion methods.^22^, ^24^, ^32^ The comparison between the images from established 4DCT‐ventilaiton and the novel 4DCT‐ventilation/perfusion methods was carried out by (1) qualitatively (using radiologist observations) and quantitatively comparing the novel images against the established methods of generating 4DCT‐ventilation, (2) comparing both the novel and established 4DCT‐based lung function images against SPECT ventilation/perfusion scans, (3) comparing functional contours generated with each type of imaging modality and (4) comparing dose‐function metrics^12^ generated with each imaging modality.

For each patient, four types of 4DCT‐ventilation/perfusion images were generated: (1) 4DCT‐ventilation based on established methods (referred to as “4DCT‐vent‐HU”),^30^ (2) 4DCT‐ventilation based on the Integrated Jacobian Formulation (referred to as “4DCT‐vent‐IJF”),^22^ (3) 4DCT‐ventilation based on the Mass Conserving Volume Change formulation (referred to as 4DCT‐vent‐MCVC’),^32^ and (4) 4DCT‐perfusion based on the mass conservation model (referred to as “4DCT‐perf”).^24^

4DCT‐vent‐HU has been well described, validated against other forms of functional imaging,^33^ and used in a prospective clinical trial.^10^ Briefly, generating 4DCT‐vent‐HU images involves advanced segmentation of the lungs (including the exclusion of airways and pulmonary vasculature) on the maximum‐inhalation and maximum‐exhalation phases of the 4DCT, DIR to link lung voxel elements from inhale to exhale, and the application of a density‐change‐based equation.^30^, ^34^ The density‐change‐based equation is derived from the assumption that CT voxel content is composed of a linear combination of water‐like material with an HU value of 0 and air‐like material with an HU Value of–1000 between breathing phases.^35^

The 4DCT‐vent‐IJF technique generates ventilation images by calculating regional volume changes using the hit‐or‐miss sampling algorithm for integral approximation, taking into account the resolution of the digital grid, and using approximations that have quantitatively defined and manageable levels of uncertainty. From the regional estimates, a Jacobian image is numerically recovered through the solution of a basic constrained linear least squares problem, ensuring that the retrieved global volume change matches the global volume change obtained from the inhale and exhale lung segmentation masks.^22^ 4DCT‐vent‐IJF methods have been shown to be more robust to variations in DIR solutions (and therefore more reproducible) when compared to traditional 4DCT‐vent‐HU or 4DCT‐ventilation Jacobian approaches.^22^

4DCT‐vent‐MCVC generates ventilation images by assuming that mass is conserved between the inhale and exhale phases of the 4DCT. 4DCT‐vent‐MCVC estimates the Jacobian factor of the DIR transformation using HU‐defined density values within a series of spatially corresponding lung subregions. Subregional volume change in 4DCT‐vent‐MCVC is estimated based on the mean density value within the subregional volume and the mean density value of the voxels mapped into the subregional volume by the DIR solution.^32^, ^36^ The full 4DCT‐vent‐MCVC image, providing volume change values for each lung voxel, is obtained from the subregional estimates by solving an inequality‐constrained linear‐least‐squares problem. Similar to the 4DCT‐vent‐IJF formulation, 4DCT‐vent‐MCVC provides improved numerical robustness compared to traditional 4DCT‐vent‐HU approaches.

The generation of 4DCT‐perf images relies on a mass‐conservation model, describing the unknown mass change as a linear combination of spatially corresponding inhale and exhale HU‐estimated voxel densities. The 4DCT‐perf technique necessitates the DIR transformation between inhale and exhale phases, preprocessing lung volume segmentation, and an estimate for the Jacobian of the DIR transformation. 4DCT‐perf is driven by the magnitude of mass changes for each voxel in the lung volume, which is formulated as the solution to a constrained linear‐least‐squares problem based on subregional mean‐magnitude mass change measurements.^24^ Similar to 4DCT‐vent‐IJF and 4DCT‐vent‐MCVC, the amount of uncertainty in a subregional sample mean measurement is related to measurement resolution and can be characterized with respect to a tolerance parameter.

### 2.3 4DCT ventilation/perfusion imaging comparison metrics

To evaluate the spatial agreement between the novel methods (4DCT‐vent‐IJF, 4DCT‐vent‐MCVC, 4DCT‐perf) and the established method of generating 4DCT‐ventilation images (4DCT‐vent‐HU) for each patient, a clinical assessment was carried to identify lung function defects. The defects were categorized by a board‐certified radiologist as presence or absence of a defect (scored as a binary yes/no), with and without specific attention to the location within the lung lobes. The binary assessment by the radiologists for each patient for the three novel methods was compared to 4DCT‐vent‐HU as the “ground truth.” Receiver operating characteristic (ROC) analysis, including accuracy, sensitivity, precision, and specificity is presented. The ROC analysis was performed considering the presence or absence of a defect as well as considering the location of the defect in addition to its presence/absence.

To compare the images from all 4DCT‐based imaging modalities to standard‐of‐care functional lung images, an ROC analysis was also performed comparing all four 4DCT‐based functional imaging methods against SPECT ventilation or SPECT perfusion. 4DCT‐vent‐HU, 4DCT‐vent‐IJF, 4DCT‐vent‐MCVC were compared against SPECT ventilation and 4DCT‐perf was compared against SPECT perfusion. Although SPECT ventilation/perfusion imaging has shortcomings, it is considered the current clinical standard of care for lung‐function imaging^33^ and enabled us to evaluate how the novel, robust methods compared against a clinical standard relative to the established 4DCT‐vent‐HU technique.

### 2.4 Functional contour comparison

In functional avoidance radiotherapy, a technique is employed to transform the functional image into a functional contour, which can in turn be used for treatment planning.^10^ To quantify the differences between 4DCT‐based functional imaging methods as they relate to functional avoidance, a Dice Similarity Coefficient (DSC) analysis was performed comparing functional contours generated using each 4DCT‐based imaging modality. Using previously presented methods,^17^ functional contours were generated by converting 4DCT‐vent‐HU, 4DCT‐vent‐IJF, 4DCT‐vent‐MCVC, and 4DCT‐perf images into percentile images and using **≥**25%, **≥**50%, and **≥**75% thresholds. Average DSCs are presented comparing the functional contours from 4DCT‐vent‐IJF, 4DCT‐vent‐MCVC, and 4DCT‐perf against the functional contours from 4DCT‐vent‐HU.

### 2.5 4DCT dose‐function metrics

In functional avoidance radiotherapy, dose‐volume metrics alone are insufficient. Rather, functional avoidance requires an assessment that integrates both dose and function. Dose‐function metrics represent whether the dose was deposited in functional or non‐functional portions of the lung. Dose‐function metrics were derived using the patient's functional avoidance radiation treatment plan and the 4DCT‐based functional treatment modalities using methods previous presented.^12^ We calculated functional mean lung doses (fMLD)^37^ at each significance level as the average dose within each functional contour (described above) and functional volumes of lung receiving ≥ 20 Gy (fV20) as the volumes within each functional contour that received ≥ 20 Gy. In total, 24 dose‐function metrics were calculated for each patient using four 4DCT‐based imaging modalities, three functional thresholds, and two dose metrics. Dose‐function metrics derived from 4DCT‐vent‐IJF, 4DCT‐vent‐MCVC, and 4DCT‐perf were compared against the dose‐function metrics derived from 4DCT‐vent‐HU. Results are presented as average and standard deviations. Statistical analysis was performed using a Friedman test (Table B1 presented in Appendix B) and pair‐wise post‐hoc analysis to assess the significance of the differences between dose‐function metrics from novel 4DCT‐based lung‐function techniques and the “gold‐standard” method while protecting against multiple comparisons errors.^38^ A Bonferroni correction minimized false‐positive errors due to multiple comparisons, resulting in an alpha level of 0.016 for a 95% confidence level. Thus, *p*‐values less than 0.016 indicated a significant difference in dose‐function metrics analysis. Finally, we applied a post‐hoc Wilcoxon signed‐rank test to evaluate the null hypothesis for fMLD and fV20 separately (e.g., H_0_ = no significant difference between fMLD_4DCT‐vent‐IJF_ and fMLD_4DCT‐vent‐HU_).

## 3 RESULTS

**FIGURE 2:**
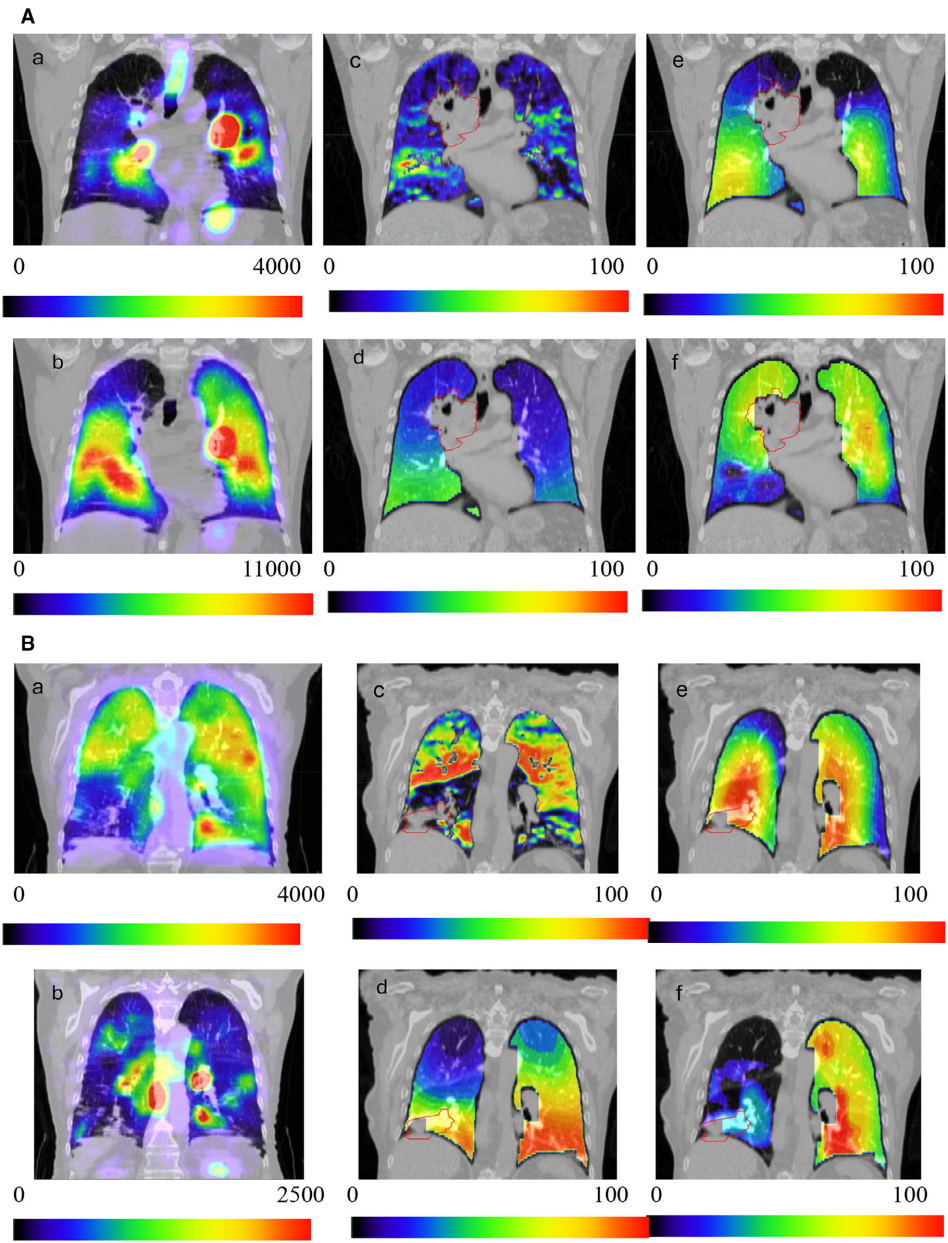
A. Representative examples of ventilation and perfusion images from the various functional imaging techniques investigated in this study. The red contour depicts the gross tumor volume (GTV). (a, and b) Ventilation and perfusion images, respectively, acquired using the standard‐of‐care SPECT technique. (c‐e) 4DCT‐ventilation images based on the Hounsfield Unit (4DCT‐vent‐HU, c), Integrated Jacobian Formulation (4DCT‐vent‐IJF, d), and Mass Conserving Volume Change (4DCT‐vent‐MCVC, e) techniques. (f) 4DCT‐based perfusion imaging (4DCT‐Perf). Figure 2‐B. Representative examples of ventilation and perfusion images from the various functional imaging techniques investigated in this study. The red contour depicts the gross tumor volume (GTV). (a, and b) Ventilation and perfusion images, respectively, acquired using the standard‐of‐care SPECT technique. (c‐e) 4DCT‐ventilation images based on the Hounsfield Unit (4DCT‐vent‐HU, c), Integrated Jacobian Formulation (4DCT‐vent‐IJF, d), and Mass Conserving Volume Change (4DCT‐vent‐MCVC, e) techniques. (f) 4DCT‐based perfusion imaging (4DCT‐Perf).

Functional lung images for a representative patient are shown in Figure 2‐A and Figure 2‐B. These figures present low/high SPECT ventilation, SPECT perfusion, 4DCT‐vent‐HU, 4DCT‐vent‐MCVC, and 4DCT‐Perf images. Qualitatively, all of the ventilation images (4a, 4c‐e) show a ventilation defect in the right‐ and left‐upper lobes. The perfusion images show homogenous perfusion except for a perfusion defect in the right upper lobe in the SPECT perfusion image and a defect in the right lower lobe in the 4DCT‐vent‐Perf image.

Table 1 shows the ROC analysis comparing the radiologist binary (yes/no of defect presence) interpretation of the novel methods (4DCT‐vent‐IJF, 4DCT‐vent‐MCVC, and 4DCT‐ Perf) against the established method of 4DCT‐vent‐HU. The data show accuracy in agreement of radiologist interpretations against 4DCT‐vent‐HU of 0.73, 0.63, 0.55, for 4DCT‐vent‐IJF, 4DCT‐vent‐MCVC, and 4DCT‐Perf, respectively. When the location of the defect was taken into account for the radiologist interpretation, 4DCT‐vent‐MCVC had the highest accuracy of 0.42 (Table A1 presented in Appendix A).

**TABLE 1:** ROC analysis of radiologist binary interpretation of novel versus classical techniques.

	4DCT‐vent‐IJF	4DCT‐vent‐MCVC	4DCT‐ Perf
Sensitivity	0.86	0.66	0.58
Precision	0.72	0.68	0.60
Specificity	0.56	0.60	0.60
Accuracy	0.73	0.63	0.55

Table 2 shows the ROC analysis comparing the radiologist binary interpretation comparing 4DCT‐vent‐HU, 4DCT‐vent‐IJF, and 4DCT‐vent‐MCVC against SPECT ventilation and 4DCT‐perf against SPECT perfusion. The data show accuracy of 0.65, 0.53, 0.58 for 4DCT‐vent‐HU, 4DCT‐vent‐IJF, and 4DCT‐vent‐MCVC, respectively and an accuracy of 0.52 when comparing 4DCT‐perf against SPECT perfusion. When the location of the defect was taken into account for the radiologist interpretation, 4DCT‐vent‐HU and 4DCT‐vent‐MCVC had the highest accuracy of 0.48 and 0.42 respectively (Table A2 in Appendix A).

**TABLE 2:** Performance comparison of radiologist binary interpretation of novel, 4DCT‐Ventilation/4DCT‐perfusion metrics with Standard SPECT Ventilation/Perfusion modalities.

	4DCT‐vent‐HU	4DCT‐vent‐IJF	4DCT‐vent‐MCVC	4DCT‐perf
**Sensitivity**	0.74	0.72	0.64	0.57
**Precision**	0.60	0.49	0.53	0.43
**Specificity**	0.58	0.38	0.53	0.49
**Accuracy**	0.65	0.53	0.58	0.52

**FIGURE 3:**
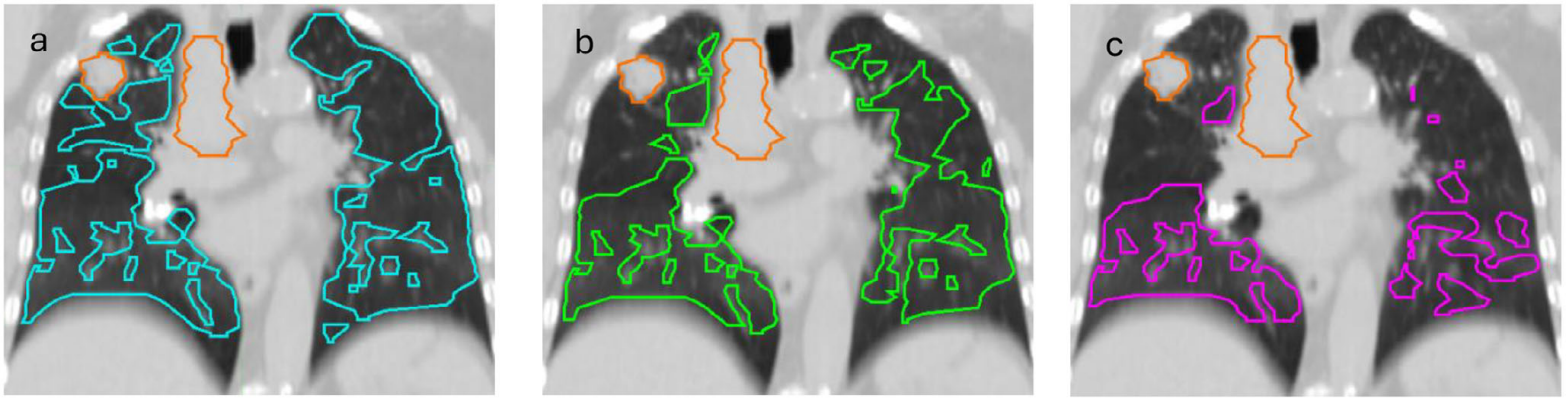
Functional contours based on the 25th, 50th, and 75th percentile thresholds for 4DCT‐vent‐HU. The gross tumor volume is outlined in Orange. (a) functional volume defined by the 25th percentile level outlined in blue, (b) functional volume defined by the 50th percentile level outlined in green, and (c) functional volume defined by the 75th percentile level outlined in pink.

Figure 3 illustrates representative contours for the 25th, 50th, and 75th percentile functional volumes for 4DCT‐vent‐HU. Qualitatively the ≥25th contours would represent the majority of the lung, while the ≥75th percentile contours would represent smaller portions of functioning voxels. Table 3 shows the DSC comparing contours based on the 25th, 50th, and 75th percentile thresholds for each of the novel methods against 4DCT‐vent‐HU. Overall, the DSC values are similar with average DSC of 0.41 ± 0.19, 0.44 ± 0.16, and 0.42 ± 0.17 for 4DCT‐vent‐IJF, 4DCT‐vent‐MCVC, and 4DCT‐Perf, respectively. The DSC was the highest, on average, for the 25th percentile contours (average DSC: 0.61) and lowest (average DSC: 0.23) for the ≥ 75th percentile contours.

**TABLE 3:** Dice similarity coefficients of novel 4DCT‐ventilation metrics against 4DCT‐ventilation Hounsfield‐Unit (4DCT‐vent‐HU).

		4DCT‐vent‐IJF	4DCT‐vent‐MCVC	4DCT‐Perf	AVG
**≥25%**	**avg **± std **range**	0.61 ± 0.06 (0.48–0.77)	0.62 ± 0.06 (0.47–0.77)	0.62 ± 0.06 (0.38–0.74)	**0.61 ± 0.06**
**≥50%**	**avg** **range**	0.41 ± 0.10 (0.13–0.69)	0.44 ± 0.08 (0.26–0.65)	0.41 ± 0.07 (0.27–0.64)	**0.42 ± 0.09**
**≥75%**	**avg** **range**	0.21 ± 0.11 (0.02–0.58)	0.26 ± 0.09 (0.04–0.54)	0.22 ± 0.07 (0.08–042)	**0.23 ± 0.09**
**avg**		**0.41 ± 0.19**	**0.44 ± 0.16**	**0.42 ± 0.17**	

**FIGURE 4:**
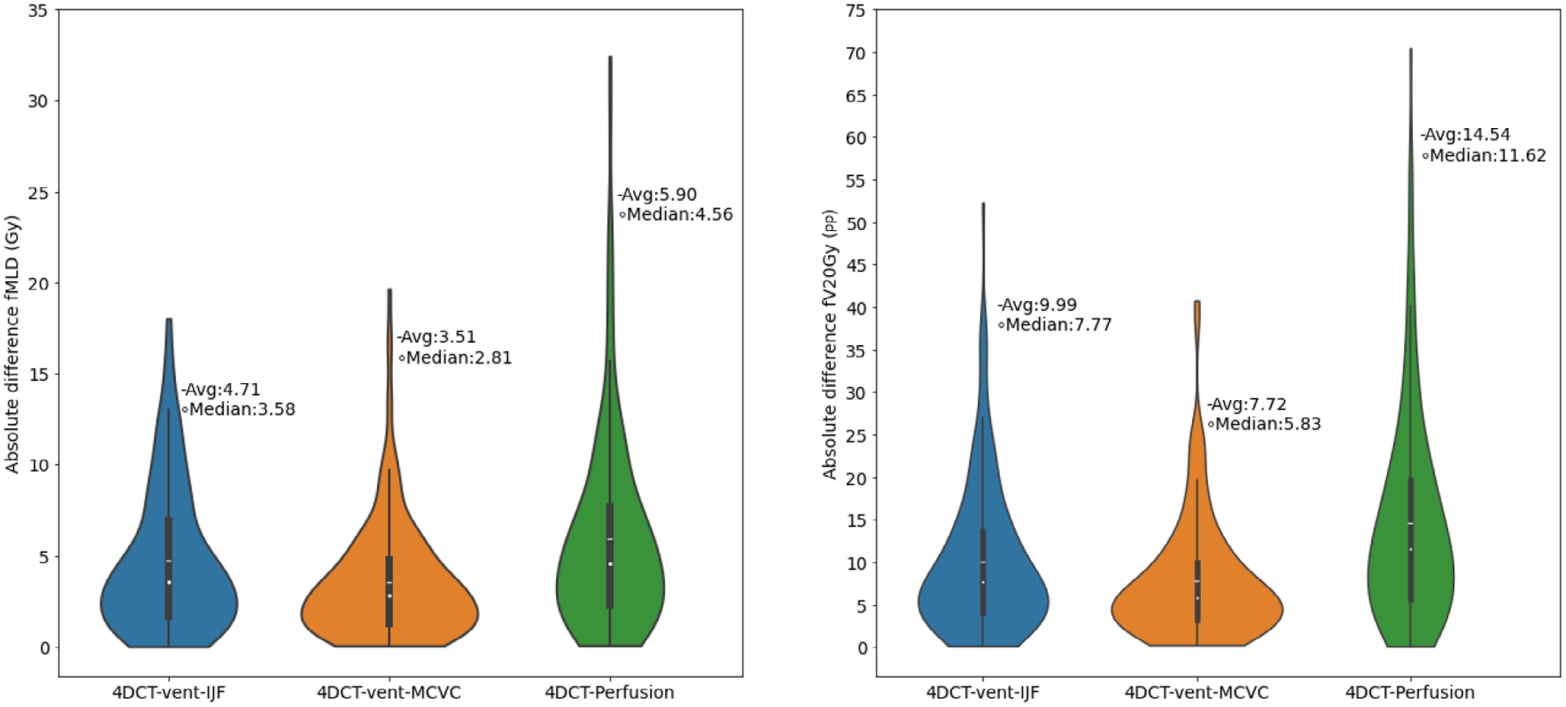
Violin plots showing the absolute differences in fMLD (Gy) and fV20 percentage point between the Hounsfield Unit‐based functional imaging technique (4DCT‐vent‐HU) and each novel 4DCT‐based technique. Novel techniques include the integral jacobian formulation (4DCT‐vent‐IJF), the mass conserving volume change (4DCT‐vent‐MCVC), and the 4DCT‐based perfusion (4DCT‐Perf).

Figure 4 shows violin plots of the differences in dose‐function measures (i.e., percentile averages of fMLD and fV20) between 4DCT‐vent‐HU images and the images from novel imaging methods (4DCT‐vent‐IJF, 4DCT‐vent‐MCVC, and 4DCT‐perf). The differences between 4DCT‐perf and 4DCT‐vent‐HU were larger than the differences between 4DCT‐vent‐MCVC, 4DCT‐vent‐IJF, and 4DCT‐vent‐HU. Table 4 shows the differences between the dose‐function metrics generated with each novel imaging method and 4DCT‐vent‐HU for functional volumes defined by all percentile thresholds (i.e., 25th, 50th, and 75th). All three novel imaging methods showed large differences in dose‐function metrics compared to those of 4DCT‐vent‐HU. The smallest difference in dose‐function metrics was in the fMLD between 4DCT‐vent‐MCVC and 4DCT‐vent‐HU (average of 3.51 ± 3.20 Gy across all functional volumes) and the largest difference was in the fMLD between 4DCT‐Perf and 4DCT‐vent‐HU (average of 5. 90 ± 5.29 Gy across all functional volumes). Within a given functional imaging modality, the largest differences occurred in the contours with the highest threshold (for example, fV20 of 13.91% for the ≥75% threshold for 4DCT‐vent‐IJF) and the smallest difference was between the measures in the contours with the lowest threshold (for example, 6.73% for the ≥25% threshold for fV20 for 4DCT‐vent‐IJF.

**TABLE 4:** Dose‐function differences between 4DCT‐ventilation metrics against 4DCT‐ventilation Hounsfield‐Unit (4DCT‐vent‐HU).

		4DCT‐vent‐IJF		4DCT‐vent‐MCVC		4DCT‐Perf	
		fMLD (Gy)	fV20 (%)	fMLD (Gy)	fV20 (%)	fMLD (Gy)	fV20 (%)
**≥25%**	**avg **± std **range** ***p*‐value**	2.85 ± 2.40 (0.1–8.6) *p* = 0.15	6.73 ± 5.02 (0.3–20.5) ‐	2.53 ± 1.82 (0.1–9.2) *p* < 0.001	5.63 ± 4.09 (0.3–21.9) ‐	3.52 ± 2.20 (0.1–9.6) *p* < 0.001	8.54 ± 5.36 (0.1–19.4) ‐
**≥50%**	**avg **± std **range** ***p*‐value**	4.38 ± 3.43 (0.0–12.9) *p* = 0.54	9.38 ± 7.56 (0.2–38.9) *p* = 0.45	3.23 ± 2.58 (0.1–10.4) *p* = 0.02	7.21 ± 5.45 (0.4–24.3) *p* = 0.23	5.91 ± 4.02 (0.0–18.7) *p* < 0.001	14.08 ± 9.62 (0.1–41.1) *p* = 0.02
**≥75%**	**avg **± std **range** ***p*‐value**	6.93 ± 4.61 (0.3–18.0) *p* < 0.01	13.91 ± 10.76 (0.7–52.3) *p* < 0.01	4.76 ± 4.30 (0.1–19.7) *p* = 0.07	10.30 ± 9.58 (0.4–40.7) *p* = 0.65	8.26 ± 7.24 (0.3–32.4) *p* < 0.001	20.27 ± 16.24 (0.1–70.4) *p* < 0.01
**avg**		4.71 ± 3.96	9.99 ± 8.61	3.51 ± 3.20	7.72 ± 7.02	5.90 ± 5.29	14.54 ± 12.25

## 4 DISCUSSION

4DCT‐ventilation has been well researched in the functional imaging field and has been clinically integrated in prospective functional avoidance clinical trials.^39^, ^40^ Despite the clinical integration and the demonstration of the clinical efficacy of 4DCT‐ventilation, studies have shown shortcomings of the integrated methods including lack of numerical robustness and sub‐optimal validation against nuclear medicine imaging.^17^, ^21^ Novel methods have been developed that have been demonstrated to improve image robustness, agreement with nuclear medicine, and provide 4DCT‐perfusion information in addition to 4DCT‐ventilation.^22^, ^23^, ^24^ Although the mathematical framework and initial validation efforts have been presented for the advanced methods, there have been no studies characterizing the clinical differences between the novel methods and the established methods of generating 4DCT‐ventilation images. The current work performed a comprehensive clinical comparison of the novel methods, 4DCT‐vent‐IJF, 4DCT‐vent‐MCFC, and 4DCT‐Perf against the established method of generating 4DCT‐ventilatin images (4DCT‐vent‐HU). Overall, our data indicate substantial differences between the novel methods and 4DCT‐vent‐HU. The accuracy when comparing radiologist interpretation of the novel methods to the radiologist interpretation of ventilation or perfusion defects ranged from 0.55 to 0.73 (Table 1). When comparing the functional contours generated from the 4DCT‐ventilation and 4DCT‐perfusion images, the results showed average DSC between 0.41 and 0.44 (Table 3). When the functional contours were extended to dose‐function metrics, differences between the novel methods and 4DCT‐vent‐HU were on the order of 2–8 Gy in fMLD and 6%–20% in fV20 (Table 4). Taken together, these data demonstrate that the novel functional imaging methods result in varying clinical interpretations, functional contours, and different dose‐function metrics.

The presented data can be evaluated in the context of previous studies comparing different functional imaging modalities. Vinogradskiy et al compared radiologist assessments of 4DCT‐ventilation against nuclear medicine planar ventilation‐perfusion scans and reported accuracy results of 81%.^33^ The 81% agreement in radiologist interpretations between 4DCT‐ventilation and nuclear medicine shows increased accuracy compared to the 55% to 73% accuracy results presented in the current work. Our data show DSC of 0.41 to 0.44, which is in line with the DSC data presented by Kipritidis et al.^17^ reporting DSC values from 0.27 to 0.73 comparing 4DCT‐ventilaiton to other forms of lung function images. Finally, the dose‐function metric differences can be compared to studies that compare functional avoidance treatment plans against standard thoracic treatment plans which have reported reductions in dose to functional lung metrics on the order of 3%–9% in functional V20^26^, ^41^, ^42^, ^43^, ^44^, ^45^ and reductions in mean dose to functional lung from 0 Gy up to 8.2 Gy.^26^, ^46^, ^47^, ^48^ The comparison to the existing data underscores the differences in the novel methods when compared to existing 4DCT‐ventilation approaches.

Although image robustness, validation, and ventilation and perfusion differences have been demonstrated between the novel 4DCT‐based lung function methods and established techniques,^22^, ^23^, ^24^ questions remained on whether using the new methods would impact functional avoidance radiotherapy treatment planning. The impact of our work is that based on the radiologist interpretations (Table 1), functional contour comparisons (Table 3), and dose‐function metric comparison (Table 4), our data indicate that functional avoidance radiotherapy would result in treatment planning and potential clinical outcome differences when compared to functional avoidance using standard (4DCT‐vent‐HU) approaches. Future work will further isolate the differences in the functional imaging methods by generating functional avoidance treatment plans using the different functional imaging methods and evaluating which combination of dose and functional imaging metrics are the best predictors of toxicity.

Several limitations exist in our work. Although the data came from a prospective clinical trial, the relatively small sample size of 63 patients may limit the generalizability of the findings. In addition, while our study included various 4DCT‐based functional imaging techniques, other emerging methods such as deep learning–based ventilation estimation^49^, ^50^ were not evaluated. The current study focused on isolating image, functional contour, and dose‐function differences between the 4DCT‐based functional imaging methods and did not evaluate the effects of clinical implementation in functional avoidance therapy via clinical (radiation toxicity following treatment) or treatment planning differences.^51^ Future studies will extend the current work by assessing how the differences in functional imaging methods extend to treatment planning and clinical toxicity alterations for functional avoidance radiation therapy.

## 5 CONCLUSION

The current work performed a comprehensive clinical comparison of novel, 4DCT‐based lung function imaging methods, 4DCT‐vent‐IJF, 4DCT‐vent‐MCFC, and 4DCT‐Perf, against the established method of generating 4DCT‐ventilatin images (4DCT‐vent‐HU). Overall, our data indicate substantial differences between the novel methods and 4DCT‐vent‐HU including radiologist observation accuracy ranging from 55% to 73%, DSC of 0.41 to 0.44, and fMLD differences from 2–8 Gy. Our data indicate that using the novel functional imaging approaches would result in different treatment planning priorities for functional avoidance radiotherapy and highlights the need for further treatment planning and clinical outcomes investigations.

## AUTHOR CONTRIBUTIONS

Ehsan Golkar wrote the first draft of the manuscript, performed MATLAB coding, and analyzed the results. Taindra Neupane performed MATLAB coding and analyzed the results. Lydia Wilson analyzed the results and reviewed the manuscript. Jennifer Kwak analyzed images and reviewed the manuscript. Richard Castillo, Edward Castillo contributed to MATLAB programming and reviewed the manuscript. Yevgeniy Vinogradskiy provided overall guidance and leadership of the study through discussions with the other authors, reviewed the results, and reviewed the manuscript.

## CONFLICT OF INTEREST STATEMENT

The authors declare no conflicts of interest.

## APPENDIX A

### A.1

Receiver operating characteristic (ROC) analysis was performed considering the presence or absence of a defect, as identified by the radiologist, by considering the location of the defect. Table A1 depicts the ROC analysis of novel methods against HU‐based 4DCT‐ventilation (4DCT‐vent‐HU). In contrast, Table A2 shows the ROC analysis comparing radiologist interpretations of 4DCT‐based functional images to those of standard SPECT ventilation/perfusion modalities.

**TABLE A1:** ROC analysis of functional ventilation imaging against 4DCT‐vent‐HU interpreted by location of lung defect.

	4DCT‐vent‐IJF	4DCT‐vent‐MCVC	4DCT‐vent‐Perf
**Sensitivity**	0.60	0.45	0.23
**Precision**	0.18	0.29	0.13
**Specificity**	0.31	0.40	0.32
**Accuracy**	0.36	0.42	0.29

**TABLE A2:** Receiver operating characteristic performance comparison of novel 4DCT‐Ventilation/4DCT‐perf metrics with standard spect ventilation/perfusion modalities.

	4DCT‐vent‐HU	4DCT‐vent‐IJF	4DCT‐vent‐MCVC	4DCT‐perf
**Sensitivity**	0.58	0.61	0.43	0.29
**Precision**	0.29	0.30	0.23	0.13
**Specificity**	0.44	0.31	0.41	0.38
**Accuracy**	0.48	0.41	0.42	0.36

## APPENDIX B

### B.1

Statistical analysis was conducted with the Friedman test corrected for multiple comparisons. Table B1 presents the resulting *p*‐values from Friedman tests comparing dose‐function measures calculated from the functional volumes of each novel 4DCT‐ventilation to those from 4DCT‐vent‐HU. *p*‐Values represent the family‐wise result for each dose‐function measure at each percentile threshold considered. Dose‐function measures included the functional mean lung dose (fMLD) and the functional volume receiving 20 Gy or more (fV20). Percentile thresholds for defining the functional volume included the 25th, 50th, and 75th percentile lung‐function levels.

**TABLE B1:** Friedman test for differences in the dose‐function measures between at least one novel 4DCT‐based functional imaging technique and the established, Hounsfield Unit–Based technique (4DCT‐vent‐HU).

	fMLD	fV20
**≥25%**	*p* < 0.001	*p* = 0.02
**≥50%**	*p* < 0.001	*p* < 0.01
**≥75%**	*p* < 0.001	*p* < 0.001
